# Sustained effectiveness of a multifaceted intervention to reduce potentially inappropriate prescribing in older patients in primary care (OPTI-SCRIPT study)

**DOI:** 10.1186/s13012-016-0442-2

**Published:** 2016-06-02

**Authors:** Barbara Clyne, Susan M. Smith, Carmel M. Hughes, Fiona Boland, Janine A. Cooper, Tom Fahey, on behalf of the OPTI-SCRIPT study team

**Affiliations:** 1 HRB Centre for Primary Care Research, Department of General Practice, Royal College of Surgeons in Ireland (RCSI), 123 St. Stephens Green, Dublin 2, Republic of Ireland; 2 School of Pharmacy, Queen’s University Belfast (QUB), 97 Lisburn Road, Belfast, BT9 7BL Northern Ireland

**Keywords:** Randomised controlled trial, Potentially inappropriate prescribing, Primary health care

## Abstract

**Background:**

Potentially inappropriate prescribing (PIP) is common in older people in primary care and can result in increased morbidity, adverse drug events and hospitalisations. We previously demonstrated the success of a multifaceted intervention in decreasing PIP in primary care in a cluster randomised controlled trial (RCT).

**Objective:**

We sought to determine whether the improvement in PIP in the short term was sustained at 1-year follow-up.

**Methods:**

A cluster RCT was conducted with 21 GP practices and 196 patients (aged ≥70) with PIP in Irish primary care. Intervention participants received a complex multifaceted intervention incorporating academic detailing, medicine review with web-based pharmaceutical treatment algorithms that provide recommended alternative treatment options, and tailored patient information leaflets. Control practices delivered usual care and received simple, patient-level PIP feedback. Primary outcomes were the proportion of patients with PIP and the mean number of potentially inappropriate prescriptions at 1-year follow-up. Intention-to-treat analysis using random effects regression was used.

**Results:**

All 21 GP practices and 186 (95 %) patients were followed up. We found that at 1-year follow-up, the significant reduction in the odds of PIP exposure achieved during the intervention was sustained after its discontinuation (adjusted OR 0.28, 95 % CI 0.11 to 0.76, *P* = 0.01). Intervention participants had significantly lower odds of having a potentially inappropriate proton pump inhibitor compared to controls (adjusted OR 0.40, 95 % CI 0.17 to 0.94, *P* = 0.04).

**Conclusion:**

The significant reduction in the odds of PIP achieved during the intervention was sustained after its discontinuation. These results indicate that improvements in prescribing quality can be maintained over time.

**Trial registration:**

Current controlled trials ISRCTN41694007.

## Introduction

Medication use in older people can improve well-being and quality of life; however, drug-related problems such as medication errors and adverse drug events (ADEs) are common [1]. Evidence suggests that prescribing in this population can be potentially inappropriate [2]. Medications are termed potentially inappropriate where their risks outweigh the benefits and when a safer therapeutic alternative is available [3]. Potentially inappropriate prescribing (PIP) is estimated to affect between 10 and 50 % of community dwelling older people internationally, increasing the risk of morbidity, ADEs, hospitalisations and health expenditure in this population [2, 4–8].

Interventions such as computerised decision support systems (CDSS), pharmacist interventions and multifaceted interventions may be useful strategies in reducing PIP in different health care settings [9–12]. We have previously demonstrated that a multifaceted intervention was effective in decreasing PIP in older patients in primary care using a short-term follow-up, on intervention completion at 4–6 months [13]. The short-term results indicated that patients in the intervention group had significantly lower odds of having PIP than patients in the control group (adjusted odds ratio (OR) 0.32, 95 % confidence interval (CI) 0.15 to 0.70, *P* = 0.02). The mean number of PIP drugs in intervention was 0.70, compared to 1.18 in control (*P* = 0.02). The intervention was effective in reducing proton pump inhibitor prescribing (adjusted OR 0.30, 95 % CI 0.14 to 0.68, *P* = 0.04), but not other drug classes [13].

The use of such short-term follow-up is a common criticism, raising concerns about the long-term sustainability of such interventions [2]. Even when inappropriate medications are ceased, evidence indicates that they might be restarted, particularly where multiple prescribers are involved [14]. Post-trial follow-up is therefore recommended to assess if short-term changes persist. Post-trial follow-up is necessary to assess if trial effects diminish, remain constant or increase after the randomised interventions are formally discontinued. The objective of this study was to determine whether the immediate improvement in PIP in the short-term was sustained at 1 year follow-up.

## Methods

A cluster randomised controlled trial (RCT) was conducted in Irish primary care to alter general practitioner (GP) PIP-related prescribing. The study protocol, intervention development and short-term outcomes (intervention completion at 4–6 months) have been reported in detail previously and are summarised in brief below [13, 15, 16]. The Research Ethics Committee of the Irish College of General Practitioners (ICGP) approved the study.

### Recruitment and randomisation

A total of 65 general practices from the greater Dublin area were invited to participate in this study with 21 (32 %) consenting. Consenting practices were assisted by the study team in identifying and recruiting approximately 10 patients per practice. Patients were eligible where they were aged ≥70 years and had pre-existing PIP (as determined by having one or more pre-specified PIP indicators, see Appendix [16]). In total, 196 patients were recruited. Fifty-three per cent of the pre-specified indicators were present in this population. Practices were allocated using minimisation to intervention or control after baseline data collection. It was not possible to blind patients or GPs to allocations; however, the outcome assessor was blinded.

### Intervention and control groups

The intervention group (11 practices, 99 patients) received a multifaceted intervention involving academic detailing with a pharmacist on how to conduct GP-led medicines review with participating patients. Medicine reviews were supported by web-based pharmaceutical treatment algorithms for GPs providing evidence-based alternative treatment options to PIP drugs and tailored patient information leaflets [15]. The control group (10 practices, 97 patients) delivered usual care and received one-off simple patient-level PIP feedback (see Table 1).

Formal support for the intervention finished at 6 months (intervention completion), and all practices (intervention and control) received a report summarising participating patients and their PIP profile for use for internal audit purposes. GPs and patients returned to their usual practice, with no attempt to encourage further medicine review or alteration to medications.

### Outcomes and statistical analysis

Outcome data were collected at 1-year post-intervention completion (i.e. 1 year after formal support for the intervention stopped). Patient records were used to collect outcome data, i.e. medication and health service use data for all eligible participants. Data was extracted by review of the patient’s chart (either electronic or paper based depending on the practice system).

The primary outcomes were the proportion of patients with PIP and the mean number of PIP drugs. The proportion of patients with PIP is presented and was analysed using a random effects logistic regression with the individual as the unit of analysis and the practice included as the random effect to control for the effects of clustering. Baseline covariates (age, gender, baseline number of PIP drugs, baseline number of repeat medications) and minimisation factors (number of GPs, practice location) were included in the model. The mean number of PIP drugs was calculated per group, and a mean difference calculated using a cluster level *t* test. Intention-to-treat analysis using random effects regression was used.

Secondary outcomes assessed differences between intervention and control in relation to individual drugs (using random effects logistic regressions) and health service utilisation including the number of GP visits and in-patient days (using random effects multiple regressions).

## Results

**Fig. 1:**
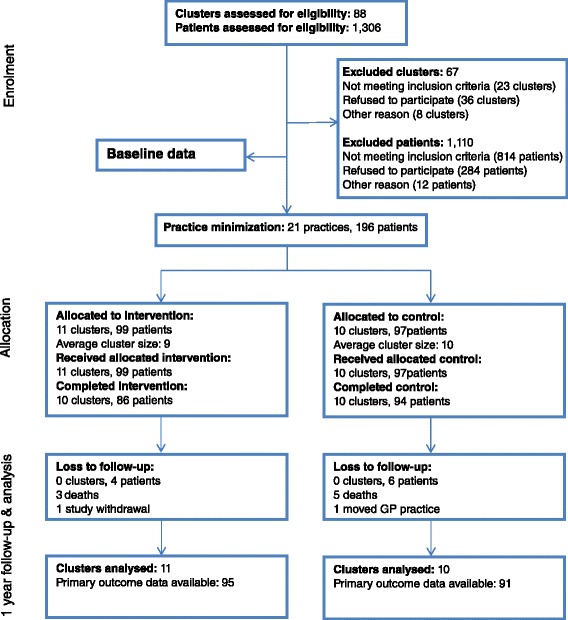
Flow of practices and patients through study

Figure 1 displays the flow of participants through the RCT. All GP practices and 186 (95 %) patients were followed up at 1 year. At baseline, receipt of proton pump inhibitors at maximum therapeutic dosage for more than 8 weeks was the most frequently occurring PIP, with 60 % of participants having this indicator [13].

### Primary outcomes

At 1-year follow-up, the proportion of patients with PIP drugs was 0.51 in the intervention group compared to 0.76 in the control group. Intervention group participants had significantly lower odds of having PIP than control participants (adjusted OR 0.28, 95 % CI 0.11 to 0.76, *P* = 0.01) (Table 2). The mean number of PIP drugs in the intervention group was 0.61 (SD 0.7) compared to 1.03 (SD 0.8) in the control group (*P* = 0.01). Intervention participants had significantly lower odds of having a potentially inappropriate proton pump inhibitor compared to controls (adjusted OR 0.40, 95 % CI 0.17 to 0.94, *s* = 0.04). No statistically significant differences were found for other drug-specific outcomes.

**Table 2:** Proportion of patients with PIP at 1-year follow-up

Characteristic	Intervention *N* (%)	Control *N* (%)	Adjusted^a^ odds ratio (95 % CI)	*P* value
PIP at baseline	99 (100)	97 (100)		
PIP at 1-year follow-up	51 (51)	74 (76)		
No PIP 1-year follow-up	48 (49)	23 (24)	0.28 (0.11 to 0.76)	0.01

^a^Adjusted for baseline number of PIP, age, gender, number of GPs in practice, practice location

### New PIP

Between baseline and 1-year follow-up, a total of 34 new instances of PIP were identified in 30 patients (13 % of total sample). In the intervention group, 12 (13 %) participants had a total of 16 new instances of PIP, compared to 18 (20 %) participants with 18 new instances of PIP in the control group (*P* = 0.38). The majority (81.2 %) had only 1 new PIP. The majority of new prescriptions identified as potentially inappropriate were proton pump inhibitors (44.1 %).

### Health service utilisation

In terms of health service utilisation, there were no statistically significant differences between intervention and control groups. Patients in the control group had an average of 11.7 GP visits compared to 12.2 in the intervention group over the 12-month period. Just over 20 % of intervention and control groups had an in-patient stay.

## Discussion

Using 1-year follow-up, we demonstrated that the significant decreases in PIP rates achieved during our intervention were sustained once it was discontinued. Our findings substantiate previous findings that have demonstrated that interventions lasting for a limited time period (e.g. educational and multifaceted interventions) can have a long-lasting carry-over effect on improving PIP [17, 18].

An array of factors may have contributed to the sustained effect observed in this study. Firstly, this cohort of older patients experienced few hospitalisations over the 1-year follow-up period, reducing the potential for medication changes or potential errors to arise at these transitions of care (i.e. moving between primary and secondary care) [19]. Hence, prescribing may have been more likely to stay stable over time. Secondly, the effect may also have been maintained due to the medications in question, particularly prescribing of proton pump inhibitors, which may be easier to maintain than other medications. Finally, the intervention itself may be an influential factor. Patients were identified as having PIP which would have been noted in the patient health care record; therefore, at future consultations, GPs may be more cognisant of not restarting the identified PIP. A combination of these factors may have contributed to the persistence of the effect from this one-off intervention.

The OPTI-SCRIPT intervention primarily affected potentially inappropriate proton pump inhibitor prescribing, which was highly prevalent at baseline (60 %). We found no impact on other included medications (e.g. benzodiazepines), likely because of the small numbers of patients exposed to these PIP drugs in this study. The prescription of inappropriate proton pump inhibitor is a substantial driver for the prevalence of PIP both in Ireland [20] and internationally [21–24]. The use of proton pump inhibitors has increased substantially during the past decade internationally, potentially due to increased long-term use for ulcer prophylaxis and perceived lack of serious adverse side effects [24]. However, a significant proportion of this prescribing has been found to be inappropriate, and consequently, there has been an increased focus on reducing inappropriate use to improve patient outcomes and decrease costs [24, 25].

**Table 1:** Summary of OPTI-SCRIPT intervention and control groups

Intervention	The intervention consisted of:
	(1) Academic detailing with a pharmacist One session (30 min) where a pharmacist visited the practice to discuss PIP, medicine review and the web-based pharmaceutical treatment algorithms (2) Medicine review with web-based pharmaceutical treatment algorithms. GPs were asked to conduct one eview per patient using the web-based platform to guide them through the process. The GP was presented with the specific PIP drug(s) for each patient, and for each PIP drug, there was a treatment algorithm with the following structure: a. The individual PIP with reason for concern b. Alternative pharmacological and non-pharmacological treatment options c. Background information (where relevant)
	(3) Patient information leaflets to give to patients during the review. Each leaflet: a. Described the PIP and the reasons as to why it may be inappropriate b. Outlined the alternative pharmacological and non-pharmacological therapies GPs may offer
Control	Control practices delivered usual care. Usual care for public general medical services (GMS) patients allows GPs to give a prescription on a monthly or three monthly basis. Control practices received simple patient-level PIP postal feedback in the form of a list summarising the medication class to which the individual patient’s potentially inappropriate medication belonged. Control practices did not receive an academic detailing visit or were not prompted to carry out medicines review with the individual patients.

*GMS* general medical services, *PIP* potentially inappropriate prescribing

Source: Clyne et al. [13]

A small proportion of the intervention group (13 %) had new PIP at 1-year follow-up, though this was lower than those in the control group (20 %), suggesting some effect on GP prescribing in the intervention practices. The new instances of PIP in intervention practices mainly related to sustained maximal dosage of proton pump inhibitors. From the data presented here, it is unclear if the proton pump inhibitor was indeed appropriate or if it was initiated by the GP or another physician. Proton pump inhibitors initiated in hospitals are frequently continued in primary care, even when inappropriate [26].

Improvements may be observed in control group participants due to reactive effects of being studied (i.e. the possible Hawthorne effect). The control group in this study did alter their prescribing patterns slightly. This may be explained by the fact that during the intervention, they received simple feedback about their patients based on baseline data collection and a report on patient PIP at intervention completion. Feedback has been found to promote slight improvements in professional practice but is most effective when it is provided intensively [27]. In anticipation of this improvements in the control group occurring, we analysed anonymised data from the Primary Care Reimbursement Service (PCRS) pharmacy claim database of dispensed medications (a national prescribing database of GP and pharmacy claims), as a national contemporaneous comparison group. Analysis of this group highlighted that the crude odds of having PIP were lower in the OPTI-SCRIPT intervention group compared to the national comparator group [13].

The study has a number of strengths, including being conducted in ‘real-world’ practices, the low rate of attrition of from the study (primarily due to the nature of the outcome data) and the completeness of the prescription data. However, there are some limitations including the geographic restriction to a region in Ireland, limiting external validity. In all, 32 % of invited GP practices were recruited which is lower than that reported in other primary care studies [28]. The intervention was effective at decreasing the most prevalent PIP in this study, proton pump inhibitors at maximum therapeutic dosage for more than 8 weeks. Potentially inappropriate proton pump inhibitor is a problem in Ireland and internationally, indicating that this intervention could be generalizable to other settings. However, it has been argued that future studies of PIP should focus on the management of genuinely high-risk medicines (i.e. prescribing likely to lead to adverse clinical outcomes [29]), rather than global lists of potentially inappropriate medications [30, 31]. It is therefore important to establish the effectiveness of the OPTI-SCRIPT intervention in altering prescribing, other than proton pump inhibitor prescribing.

## Conclusions

Changes in PIP occur against a background of escalating polypharmacy and changes in prescribing patterns of specific medications over time [20]; however, these findings indicate that improvements in prescribing quality can be maintained over time.

## Abbreviations

CI, confidence interval; GP, general practitioner; ICGP, Irish College of General Practitioners; OR, odds ratio; PIP, potentially inappropriate prescribing; RCT, randomised controlled trial

## Appendix

**Table 3:** Selected prescribing criteria/prescribing indicator [16]

Criteria	Concern	Estimated prevalence in Ireland^a^
PPI for peptic ulcer disease at full therapeutic dosage for >8 weeks	Earlier discontinuation or dose reduction for maintenance/prophylactic treatment of peptic ulcer disease, oesophagitis or GORD indicated	4.1–16.7 %
NSAID (>3 months) for relief of mild joint pain in osteoarthritis	Simple analgesics preferable and usually as effective for pain relief	1.1–8.8 %
Long term (i.e. >1 month), long-acting benzodiazepines, e.g. chlordiazepoxide, flurazepam, nitrazepam, chlorazepate and benzodiazepines with long-acting metabolites, e.g. diazepam	Risk of prolonged sedation, confusion, impaired balance, falls	3.0–9.1 %
Any regular duplicate drug class prescription, e.g. 2 concurrent opiates, NSAIDs, SSRIs, loop diuretics, and ACE inhibitors. Excludes duplicate prescribing of drugs that may be required on a PRN basis, e.g. inhaled beta 2 agonists (long and short acting) for asthma or COPD, and opiates for management of breakthrough pain	Optimisation of monotherapy within a single drug class should be observed prior to considering a new class of drug	2.2–6.0 %
TCAs with an opiate or calcium channel blocker	Risk of severe constipation	0.4–2.0 %
Aspirin at dose >150 mg/day	Increased bleeding risk, no evidence for increased efficacy	0.1–1.0 %
Theophylline as monotherapy for COPD/asthma	Risk of adverse effects due to narrow therapeutic index	0.6–1.2 %
Use of aspirin and warfarin in combination without histamine H_2_ receptor antagonist (except cimetidine because of interaction with warfarin) or PPI	High risk of GI bleeding	0.3–1.1 %
Doses of short-acting benzodiazepines, doses greater than lorazepam (Ativan®), 3 mg; oxazepam (Serax®), 60 mg; alprazolam (Xanax®), 2 mg; temazepam (Restoril®), 15 mg; and triazolam (Halcion®), 0.25 mg	Total daily doses should rarely exceed the suggested maximums	1.0–1.5 %
Prolonged use (>1 week) of first generation antihistamines, i.e. diphenydramine, chlorpheniramine, cyclizine, promethazine	Risk of sedation and anticholinergic side effects	<1.0 %
Warfarin and NSAID together	Risk of GI bleeding	0.7–1.7 %
Calcium channel blockers with chronic constipation	May exacerbate constipation	<1.0 %
NSAID with history of peptic ulcer disease or GI bleeding, unless with concurrent histamine H_2_ receptor antagonist, PPI or misoprostol	Risk of peptic ulcer relapse	<1.0 %
Bladder antimuscarinic drugs with dementia	Risk of increased confusion, agitation	<1.0 %
TCAs with constipation	May worsen constipation	<1.0 %
Digoxin at a long-term dose >125 μg/day (with impaired renal function)	Increased risk of toxicity	<1.0 % <1.0 %
Thiazide diuretic with a history of gout	May exacerbate gout	<1.0 %
Glibenclamide (with type 2 diabetes mellitus)	Risk of prolonged hypoglycaemia	<1.0 %
Aspirin with a past history of peptic ulcer disease without histamine H2 receptor antagonist or PPI	Risk of bleeding	<1.0 %
Prochlorperazine (Stemetil®) or metoclopramide with parkinsonism	Risk of exacerbating parkinsonism	<1.0 %
TCAs with dementia	Risk of worsening cognitive impairment	<1.0 %
TCAs with glaucoma	Likely to exacerbate glaucoma	<1.0 %
TCAs with cardiac conductive abnormalities	Pro-arrhythmic effects	<1.0 %
Long-term corticosteroids (>3 months) as monotherapy for rheumatoid arthritis or osteoarthritis	Risk of major systemic corticosteroid side effects	<1.0 %
Bladder antimuscarinic drugs with chronic prostatism	Risk of urinary retention	<1.0 %
NSAID with heart failure	Risk of exacerbation of heart failure	<1.0 %
TCAs with prostatism or prior history of urinary retention	Risk of urinary retention	<1.0 %
Systemic corticosteroids instead of inhaled corticosteroids for maintenance therapy in COPD/asthma	Unnecessary exposure to long-term side effects systemic steroids	<1.0 %
Bladder antimuscarinic drugs with chronic glaucoma	Risk of acute exacerbation of glaucoma	<0.01 %
NSAID with SSRI	Increased risk of GI bleed	N/A
Bladder antimuscarinic drugs with chronic constipation	Risk of exacerbation of constipation	N/A
Prednisolone (or equivalent) >3 months or longer without bisphosphonate	Increased risk of fracture	N/A
NSAID with ACE-inhibitor	Risk of kidney failure, particularly if presence of general arteriosclerosis, dehydration or concurrent use of diuretics	N/A
NSAID with diuretic	May reduce the effect of diuretics and worsen existing heart failure	N/A

*Abbreviations*: *ACEI* angiotensin-converting-enzyme inhibitor, *COPD* chronic obstructive pulmonary disease, *GI* gastro-intestinal, *N/A* not available, *GORD* gastro-oesophageal reflux disease, *NSAID* nonsteroidal anti-inflammatory drug, *PPI* proton pump inhibitor, *PRN* Pro re nata, as needed, *SSRI* selective serotonin reuptake inhibitor, *TCA* tricyclic anti-depressant

^a^Prevalence—the proportion of the study population with 1 or more potentially inappropriate medications from the literature
